# Thrombospondin 2/Toll-Like Receptor 4 Axis Contributes to HIF-1α-Derived Glycolysis in Colorectal Cancer

**DOI:** 10.3389/fonc.2020.557730

**Published:** 2020-11-10

**Authors:** Chunjie Xu, Lei Gu, Manzila Kuerbanjiang, Siyuan Wen, Qing Xu, Hanbing Xue

**Affiliations:** ^1^Department of Gastrointestinal Surgery, Renji Hospital, School of Medicine, Shanghai Jiao Tong University, Shanghai, China; ^2^Ottwa -Shanghai Joint School of Medicine, Shanghai Jiao Tong University, Shanghai, China; ^3^Division of Gastroenterology and Hepatology, Key Laboratory of Gastroenterology and Hepatology, Ministry of Health, Renji Hospital, School of Medicine, Shanghai Jiao Tong University, Shanghai Institute of Digestive Disease, Shanghai, China

**Keywords:** colorectal cancer, thrombospondin 2, Toll-like receptor 4, aerobic glycolysis, HIF-1α

## Abstract

**Background:**

Aerobic glycolysis is a typical metabolic reprogramming in tumor cells, which contributes to the survival and proliferation of tumor cells. The underlying mechanisms controlling this metabolic switch in colorectal cancer (CRC), however, remain only partially understood.

**Methods:**

The Cancer Genome Atlas (TCGA) dataset and Gene Expression Omnibus (GEO) (GDS4382, GSE6988, GSE35834) were used to analyzed the mRNA expression of THBS2. 392 paired samples of CRC and adjacent non-cancerous tissues were collected to detect the expression of THBS2 by IHC. The correlation of THBS2 expression with categorical clinical variables in patients with CRC was evaluated using chi-square analysis or Student’s *t*-test. CCK-8, colony formation, and animal CT scan were used to functional analysis of THBS2 in CRC.

**Results:**

Thrombospondin 2 (THBS2) is aberrantly upregulated and linked to a poor prognosis in CRC. Subsequent experiments also showed that THBS2 promotes the proliferation of CRC cells. In terms of mechanism, THBS2 interacted with Toll-like receptor 4 (TLR4), but not with the other toll-like receptors (TLRs), which upregulated the mRNA expression of *GLUT1, HK2, ALDOA, PKM2*, and *LDHA* and enhanced glycolytic capacity in CRC cells. Moreover, THBS2/TLR4 axis significantly increased the protein level of HIF-1α and blocking HIF-1α by siRNA reversed the enhanced glycolytic capacity and the upregulated expression of glycolytic enzymes in CRC cells.

**Conclusion:**

Our findings revealed that the THBS2/TLR4 axis contributes to HIF-1α derived glycolysis and eventually promotes CRC progress.

## Highlights

Thrombospondin 2 (THBS2) interacts with TLR4, enhances aerobic glycolysis by regulating HIF-1α, and eventually contributes to CRC progress.

## Introduction

Colorectal cancer (CRC) is one of the most common malignant tumors and ranks third in terms of incidence and mortality associated with human carcinoma (1). Given the considerable advances in diagnosis and therapy, early-stage CRC, including stage 1 and stage 2, has a relatively good prognosis with a 5-year survival rate. However, the prognosis of stages 3 and 4 patients extremely weakens with the progress of CRC. Therefore, it is essential to explore the mechanism underlying CRC progress to design potential therapeutic strategies.

The thrombospondin (THBS) family has five members (THBS1, THBS2, THBS3, THBS4, and THBS5); the family is a disulfide-linked homotrimeric glycoprotein that mediates cell-to-cell and cell-to-matrix interactions (2). Recently, THBS has been reported to be involved in various kinds of malignant tumors, including lung cancer, gastric cancer, colorectal cancer, and liver cancer (3–6). THBS2 is a member of the THBS family, which is reported to be regulated by the microRNA network in human cancer (7–9). However, little is known about the mechanism underlying THBS2-mediated CRC progress.

Toll-like receptors (TLRs) are common pattern recognition receptors. Early studies confirm that TLRs mainly recognize exogenous pathogens and induce the innate immunity of the human body. TLRs are widely researched in human cancer, the activation of which will eventually contribute to the malignant biological behavior of tumor cells (10). Recent emerging evidence has shown that endogenous ligands or molecules produced by tumor cells interact with TLRs, which stimulates cancer progress (11). However, the mechanism underlying TLRs’ contribution to CRC progress requires further investigation.

Metabolic reprogramming is a characteristic change in tumor cells, which not only meets the energy demand for the rapid proliferation of tumor cells but also provides essential substances for tumor development (12). Therefore, metabolic reprogramming is a complex program, including glucose metabolism, fatty acid metabolism, amino acid metabolism, nucleotide metabolism, and glutamine metabolism (13). To the best of our knowledge, glucose is the principal energy substance for providing ATP. Glucose metabolism reprogramming is also widely reported, of which the Warburg effect is the most famous one. The Warburg effect means that tumor cells tend to underlie glycolysis but not oxidative phosphorylation, even in normoxia. The metabolic switch to glycolysis in tumor cells is controlled by glycolysis-related enzymes (GLUT1, LDHA, ALDOB) and vital signaling pathways (PI3K-AKT, mTOR, Myc, HIF-1α) (14).

In this study, we first found that THBS2/TLR4 interaction promotes the tumor growth of CRC by enhanced HIF-1α-mediated glycolysis. Similarly, the analyzed clinical information shows that patients with higher expression of THBS2 tend to have a larger tumor size, higher T stage, and lower survival rate. Therefore, our study may provide new therapeutic insights for patients with CRC.

## Materials and Methods

### Patients and Samples

A total of 392 paraffin sections of CRC tissues and adjacent paired non-cancerous tissues were collected to design a tissue array chip from the Department of Gastrointestinal Surgery, Renji Hospital, School of Medicine, Shanghai Jiao Tong University. All patients with CRC underwent surgery at the Department of Gastrointestinal Surgery, Renji Hospital, School of Medicine, Shanghai Jiao Tong University between January 2014 and January 2016. The study was approved by the Research Ethics Committee of Renji Hospital and carried out in accordance with the ethical standards formulated in the Helsinki Declaration. All patients provided their informed consent.

### Cell Culture

LoVo, RKO, SW480, and SW620 cells (human CRC cell lines) were obtained from the Cell Bank of the Chinese Academy of Sciences (Shanghai, China), which all performed genotyping of human cancer cell lines and had no cross-contamination. All cell lines were cultured in Dulbecco’s modified Eagle’s medium, supplemented with 10% fetal bovine serum and 1% penicillin and streptomycin.

#### Colony Assay

1×10^3^ cells were seeded into 6-well plates. After 14 days, cells were stained with 0.1% crystal violet solution and the number of colonies (>50 cells) was counted under the microscope. Each experiment was carried out independently in triplicate.

### Small Interfering RNA Transfection

The siRNAs for TLRs (TLR1-10) were purchased from GenePharma (Shanghai GenePharma Co., Ltd., Shanghai, China). **Table S1** displays the sequences, and the experimental method was performed as previously described (15).

### Lentivirus Transfection

A lentivirus was used to transfect full-length human THBS2 cDNA into CRC cell lines to generate Lentivirus-THBS2 (THBS2-OV). Lentivirus-NC was used as a negative control (THBS2-vector). In addition, one short-hairpin RNA (shRNA) sequence against THBS2 was transfected into CRC cell lines to generate shRNA-THBS2, while sh-NC-THBS2 was used as a negative control. **Table S1** shows the sequences of shRNA.

### RNA Isolation and Real-Time Quantitative Polymerase Chain Reaction

Trizol was used to extract RNA, and total RNA was reverse transcribed to cDNA by PrimeScriptTM (TAKARA). We used 18S RNA as an internal control. **Table S1** shows the sequences of the primers. The relative expression of the target gene was calculated by the −△△Ct method.

### Western Blot Analysis

The radioimmunoprecipitation assay (RIPA) buffer was used to extract total protein, supplemented with 1% protease inhibitors (P8340, Sigma-Aldrich) and phosphatase inhibitors (P5726, Sigma-Aldrich). The bicinchoninic acid (BCA) assay was used to measure protein concentration. Western blot analysis was performed, as previously described (12). THBS2 (sc-136238, Santa Cruz Biotechnology), TLR4 (ab30667, Abcam), Ki67 (Proteintech Group, Inc.), HIF-1α (ab2185, Abcam), GLUT1 (ab115730, Abcam), HK2 (ab104836, Abcam), ALDOA (ab150396, Abcam), PKM2 (ab137852, Abcam), and LDHA (ab101562, Abcam) primary antibodies were used. Horseradish peroxidase (HRP)-conjugated AffiniPure goat anti-rabbit IgG (H+L) and HRP-conjugated AffiniPure goat anti-mouse IgG (H+L) were obtained from Proteintech Group, Inc. (Jackson).

### Immunohistochemistry

All tissues were paraffin-embedded and cut into 4-m thick sections. All sections were dewaxed with xylene and hydrated with alcohol. Sodium citrate was used for antigen retrieval, and 0.3% of hydrogen peroxide (H2O2) was used to block endogenous peroxidase. After blocking non-specific sites with bovine serum albumin, all the sections were incubated with an appropriate primary and secondary antibody. We used the 3,3-diaminobenzidine (DAB) kit for visualization, and hematoxylin was used to stain nuclei. All the sections were dehydrated with alcohol and sealed with neutral resin. The IHC staining score was calculated based on pixel intensity; staining was scored as per the staining intensity: no staining, 1; weak staining, 2; moderate staining, 3; and strong staining, 4.

### Seahorse Analyses

The Seahorse XF96 Flux Analyzer (Seahorse Bioscience, Agilent) was used to carry out the extracellular acidification rate (ECAR) and oxygen consumption rate (OCR) in the CRC cell lines. Briefly, all CRC cells used in this paper, including LoVo, RKO, SW620, and SW480 cells, were seeded into an XF96-well plate. The media were replaced with assay media 1 h before the assay. For ECAR assay (Seahorse Cat.#103020-100), 10 mM glucose, 1 μM oligomycin, and 50 mM 2-deoxyglucose (2-DG) were added to the wells. For the OCR test (Seahorse Cat.#103015-100), 1 μM oligomycin, 1 μM FCCP, 0.5 μM rotenone, and 0.5 μM actinomycin A were added to the wells at a special time point. Both measurements were normalized by total protein quantitation. The above experiments were performed in triplicate and repeated twice.

### Glucose and Lactate Measurement

The Amplex^®^ Red Glucose/Glucose Oxidase Assay Kit (Invitrogen, Cat.#A22189) was used to measure the glucose uptake. Glucose consumption was calculated by the net content of the original glucose concentration deduced the measured glucose concentration in the medium. The Lactate Assay Kit (BioVision, Cat.#ABIN411683) was used to measure lactate production. Total proteins were used for the normalization of the results obtained above. These experiments were performed in triplicate and repeated twice.

### Animal Model

For the generation of an orthotopic model of CRC, all nude mice were anesthetized with 0.5% pentobarbital. After opening the abdominal cavity, 1*10^6 LoVo or SW620 cells/null mice were injected into the ileocecum. After 4 weeks, the mice were killed, and the tumor tissues were excised and weighed. All tissues were fixed with 4% paraformaldehyde. All animal experiments were approved by the Research Ethics Committee of Renji Hospital and adhered to the local and national requirements for the care and use of laboratory animals.

### Co-Immunoprecipitation

Total protein was extracted from CRC cells and incubated overnight with the appropriate primary antibody, followed by the addition of protein A-Sepharose beads. After extensive washing, the precipitates were subjected to Western blotting for the detection of the interacting proteins. Normal rabbit IgG served as a negative control. Anti-hemagglutinin was purchased from Medical & Biological Laboratories (Nagoya, Japan). Anti-THBS2 (sc-136238) was obtained from Santa Cruz Biotechnology, while anti-TLR4 (ab30667) was supplied by Abcam.

### Statistical Analysis

Measurement data are presented as the mean ± standard deviation (SD). SPSS 20.0 (Chicago, IL, USA) and GraphPad Prism 5 software were used to conduct the statistical analyses. The correlation of THBS2 expression with categorical clinical variables in patients with CRC was evaluated using chi-square analysis or Student’s *t*-test. Measurement data, such as age and tumor size, were evaluated using Student’s *t*-test, while categorical variables and ranked data, such as gender, T stage, lymph node invasion, and distant metastasis, were analyzed using the chi-square test. Spearman’s rank correlation was used for the analysis of two-way ordered categorical data. Survival curves were generated using the Kaplan–Meier method and analyzed by the log-rank test. Statistical significance was accepted at *p* < 0.05.

## Results

### Aberrantly Upregulated THBS2 Displayed a Tumor-Promoting Role in CRC

**Figure 1:**
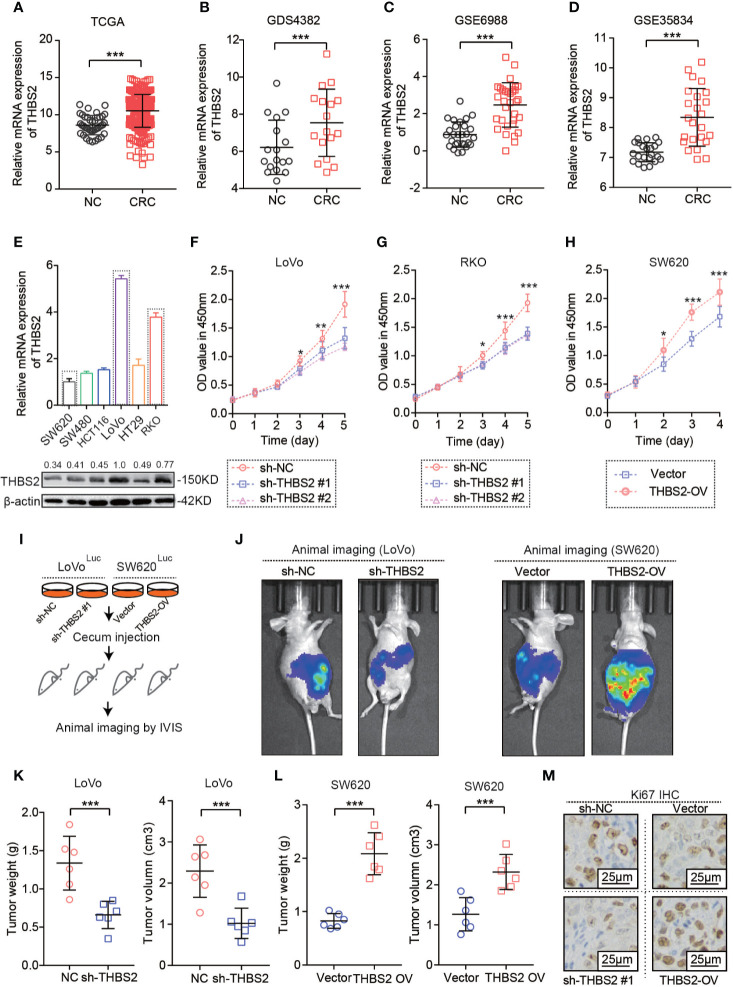
THBS2 was aberrantly upregulated and displayed a tumor-promoting role in CRC. **(A)** The mRNA expression of THBS2 in CRC and adjacent non-cancerous tissues, as analyzed from the TCGA dataset. **(B)** The mRNA expression of THBS2 in CRC and adjacent non-cancerous tissues, as analyzed from the GDS4382 dataset. **(C)** The mRNA expression of THBS2 in CRC and adjacent non-cancerous tissues, as analyzed from the GSE6988 dataset. **(D)** The mRNA expression of THBS2 in CRC and adjacent non-cancerous tissues, as analyzed from the GSE35834 dataset. **(E)** The mRNA expression of THNB2 in six CRC cell lines. **(F)** The viability of LoVo cells transfected with sh-THBS2 or sh-control, as analyzed with CCK-8 assay. **(G)** The viability of RKO cells transfected with sh-THBS2 or sh-control, as analyzed with CCK-8 assay. **(H)** The viability of SW620 cells transfected with Vector or THBS2-OV, as analyzed with CCK-8 assay. **(I)** Establishing an orthotopic tumor model by injecting tumor cells with luciferase into the cecum of BALB/c nude mice and imaging by CT. **(J)** Vivo imaging of orthotopic tumor by injecting sh-THBS2 or sh-control LoVo cell and Vector or THBS2-OV SW620 cells in the cecum (*n* = 6 in every group). **(K)** Tumor burden and Tumor volume in the cecum by injecting sh-THBS2 or sh-control LoVo cell (*n* = 6 in every group). **(L)** Tumor burden and Tumor volume in the cecum by injecting Vector or THBS2-OV SW620 cells (*n* = 6 in every group). **(M)** Expression of Ki-67 in the orthotopic tumor tissues. All experiments were performed in triplicate. Measurement data are presented as the mean ± SD. Student’s *t*-test was used for statistical analysis; *p < 0.05, **p < 0.01, ***p < 0.001.

The comprehensive analysis of THBS2 expression in CRC based on TCGA and GEO datasets showed a very significant increase in THBS2 expression in the mRNA level in CRC (**Figures 1A–D**). To verify the main function of THBS2 involved in the CRC development, we detected mRNA and protein expression of THBS2 in seven CRC cell lines and selected two cell lines with high expression of THBS2 (RKO and LoVo) and low expression of THBS2 (SW620) (**Figure 1E**). Furthermore, we knocked down the THBS2 expression in RKO and LoVo by shRNA and overexpressed it in SW620 by lentivirus (**Figures S1A–S1C**). *In vitro* experiments demonstrated that the knockdown or overexpression of THBS2 impairs or enhances the proliferation of tumor cells (**Figures 1F–H**; **Figures S1D–F**). After establishing an orthotopic tumor model through the injection of tumor cells into the cecum of BALB/c nude mice, we discovered that THBS2 knockdown or overexpression obviously inhibited and accelerated tumor growth (**Figures 1I–L**). THBS2 overexpressed orthotopic tumors showed a consistently higher staining intensity of KI-67 than that in the control of orthotopic tumors and THBS2 knock-downed orthotopic tumors showed a lower KI-67 expression than that in the control of orthotopic tumors (**Figure 1M**). Therefore, THBS2 plays a tumor-promoting role in CRC development.

### THBS2 Promotes CRC Cells Proliferation by Interacting With TLR4

**Figure 2:**
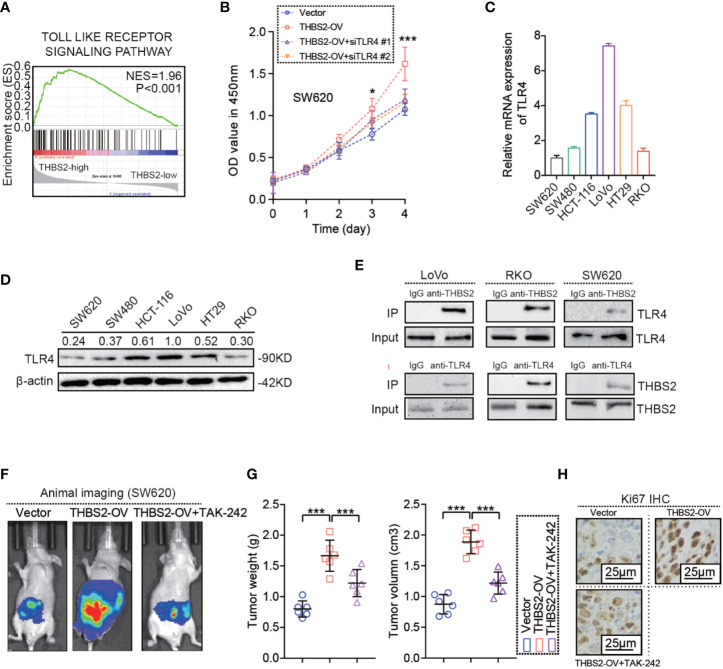
THBS2 promotes the proliferation of CRC cells by interacting with TLR4. **(A)** GSEA analysis of THBS2 mRNA expression in CRC, as evaluated from the TCGA dataset. **(B)** Viability of THBS2-OV SW620 cells transfected with si-control and si-TLR4. **(C)** mRNA expression of TLR4 in CRC cell lines. **(D)** protein expression of TLR4 in CRC cell lines. **(E)** Co-IP of THBS2 with TLR4 in LoVo, RKO and SW620 cells. **(F)** Vivo imaging of orthotopic tumor by SW620 cell in Vector, THBS2-OV and THBS2-OV+TAK-242 groups (*n* = 6 in every group), intraperitoneal injection of TAK-242 (1 μg/100 μl) in this study. **(G)** Tumor weight and volume of orthotopic tumor by SW620 cell in Vector, THBS2-OV and THBS2-OV+TAK-242 groups (*n* = 6 in every group), intraperitoneal injection of TAK-242 (1 μg/100 μl) in this study. **(H)** KI67 expression in Vector, THBS2-OV and THBS2-OV+TAK-242 groups (*n* = 6 in every group). Measurement data are presented as the mean ± SD. Student’s *t*-test was used for statistical analysis. ns. represents no statistical difference; *p < 0.05; ***p < 0.001.

As noted above, THBS2 has a tumor-promoting role in CRC. However, the underlying mechanism by which THBS2 promotes CRC development is still unknown. First, we conducted a Gene Set Enrichment Analysis (GSEA) based on the THBS2 expression of CRC samples from TCGA dataset in which all CRC samples were divided into two groups based on the expression level of THBS2, including the high expression of THBS2 group (THBS2 high group) and the low expression of THBS2 group (THBS2 low group). The result showed that the gene sets from the THBS2 high expression group are enriched in the TLR pathway (**Figure 2A**). This prompted a link between THBS2 and TLR pathway. To our surprise, THBS2 knockdown and overexpression did not affect TLRs expression (**Figures S2A–C**). However, silencing TLR4 but not the other TLRs by siRNA reversed the pro-survival role in THBS2 overexpressed CRC cells (**Figures 2B** and **S2D–L**). TLR4 expression was detected in six CRC cell lines (**Figures 2C, D**). Subsequent experiments also illustrated the interaction between THBS2 and TLR4 in CRC cells (**Figure 2E**). Moreover, the *in vivo* experiment indicated that TLR4 inhibition by a specific inhibitor (i.e., TAK-242) reversed the pro-survival role in THBS2 overexpressed CRC cells (**Figures 2F, G****)**. These results suggest that THBS2 promotes the proliferation of CRC cells by regulating the TLR4 receptor.

### THBS2/TLR4 Interaction Enhances Aerobic Glycolysis in CRC Cells

**Figure 3:**
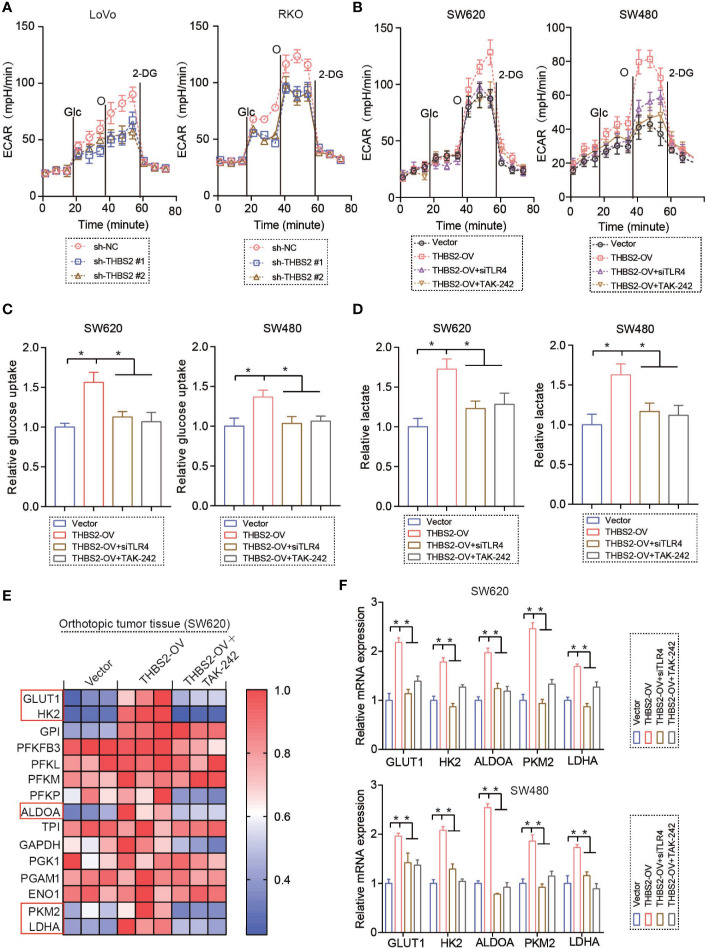
THBS2/TLR4 interaction enhances aerobic glycolysis in CRC cells. **(A)** Extracellular acidification rate (ECAR) of LoVo or RKO cells in the sh-NC and sh-THBS2 group was detected *via* a Seahorse Bioscience XFp analyzer. Glc, glucose; O, oligomycin; 2-DG, 2-deoxy-d-glucose. **(B)** Extracellular acidification rate (ECAR) of SW620 or SW480 cells in the Vector and THBS2-OV group with the treatment of siTLR4 or TAK-242 was detected *via* a Seahorse Bioscience XFp analyzer. Glc, glucose; O, oligomycin; 2-DG, 2-deoxy-d-glucose. **(C)** Glucose uptake of SW620 or SW480 cells in the Vector, THBS2-OV, THBS2-OV+ siTLR4, and THBS2-OV+ TAK-242 group. **(D)** Lactic acid formation of SW620 or SW480 cells in the Vector, THBS2-OV, THBS2-OV+siTLR4, and THBS2-OV+TAK-242 group. **(E)** mRNA expression of relative genes in glycolysis in the Vector, THBS2-OV, and THBS2-OV +TAK-242 group in orthotopic tumor tissue. **(F)** mRNA expression of relative genes in the glycolysis of SW620 or SW480 cells in Vector, THBS2-OV, THBS2-OV +siTLR4, and THBS2-OV+TAK-242 groups. 10μM TAK-242 *in vitro* experiment. Measurement data are presented as the mean ± SD. Student’s *t*-test was used for statistical analysis; *p < 0.05.

In this study, we found that THBS2 knockdown and overexpression significantly inhibit and enhance glycolysis, albeit without any obvious effect on oxidative phosphorylation in CRC cell lines (**Figures 3A, B**, **S3A**, and **S3B**). Moreover, silencing TLR4 by siRNA almost reversed the THBS2-enhanced glycolysis (**Figure 3B**). Also, THBS2 knockdown and overexpression consistently reduced and increased glucose consumption and lactate production (**Figures 3C, D**, **S3C**, and **S3D**). Silencing TLR4 almost reversed the THBS2-enhanced glucose consumption and lactate production (**Figures 3C, D**). Glycolysis was regulated by a series of related enzymes and vital signaling pathway. Consequently, THBS2 upregulated the expression of glycolysis-related genes (*GLUT1, HK2, ALDOA, PKM2*, and *LDHA*) *in vivo* and *in vitro* (**Figures 3E, F**, **S3E, F, G**, **S4A, B**). Silencing TLR4 almost reversed the upregulated *GLUT1, HK2, ALDOA, PKM2*, and *LDHA* by THBS2 overexpression (**Figures 3E, F**, and **S3G**). Moreover, THBS2/TLR4 interaction also upregulated the expression of glycolysis related genes when the cells are under hypoxic conditions (**Figures S3H**).

### THBS2/TLR4 Interaction Increases HIF-1α Expression in CRC Cells

**Figure 4:**
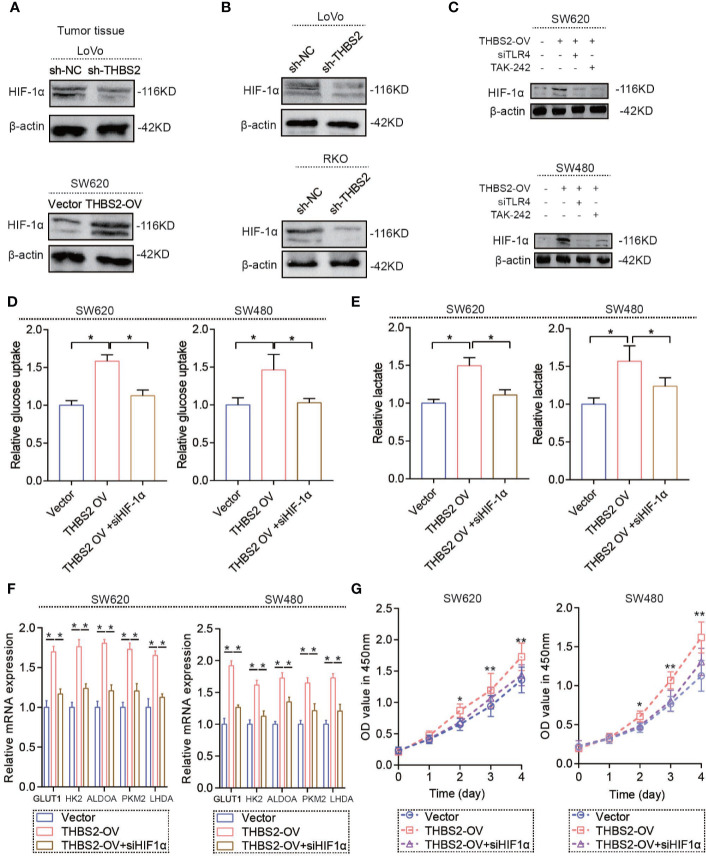
THBS2/TLR4 axis increased HIF-1α expression in CRC cells. **(A)** Protein expression of HIF-1α of LoVo cells in the sh-NC and sh-THBS2 group and SW620 cells in the Vector and THBS2-OV group. **(B)** Protein expression of HIF-1α of LoVo and RKO cells in the sh-NC and sh-THBS2 groups. **(C)** Protein expression of HIF-1α of SW620 and SW480 cells in the Vector, THBS2-OV, THBS2-OV+siTLR4, and THBS2-OV+TAK-242 group. **(D)** Glucose uptake of SW620 or SW480 cells in the Vector, THBS2-OV, THBS2-OV+HIF1α group. **(E)** Lactic acid formation of SW620 or SW480 cells in the Vector, THBS2-OV, THBS2-OV+HIF1α group. **(F)** mRNA expression of *GLUT1, HK2, ALDOA, PKM2*, and *LDHA* of SW620 and SW480 cells in the Vector, THBS2-OV, and THBS2-OV+siHIF-1α group. **(G)** The viability of SW620 or SW480 cells in the Vector, THBS2-OV, and THBS2-OV+siHIF-1α group, as analyzed with CCK-8 assay. Student’s *t*-test was used for statistical analysis. ns. represents no statistical difference; *p < 0.05, **p < 0.01.

As mentioned above, the THBS2/TLR4 axis upregulated the mRNA expression of glycolysis-related genes, which resulted in a high glycolytic state in CRC cells. However, the underlying mechanism by which the THBS2/TLR4 axis regulates the mRNA expression of glycolysis-related genes is still unknown. HIF-1α is a vital transcription factor for regulating glycolysis by transcriptionally activating a series of glycolysis-related genes. Our results indicate that THBS2 overexpression upregulates the protein expression of HIF-1α and that THBS2 knockdown inhibits the protein expression of HIF-1α *in vivo* (**Figure 4A**). THBS2/TLR4 axis did not affect the mRNA level of HIF-1α, PHD1, PHD2, and PHD3 (**Figures S4C, D**), which may suggest THBS2/TLR4 axis contributes to translation of HIF-1α. In tissue microarray, THBS2 had a significant correlation with HIF-1α (**Figure S4E**). Moreover, the *in vitro* experiment also showed THBS2 overexpression and knockdown upregulated and reduced the protein expression of HIF-1α (**Figures 4B, C**). Silencing HIF-1α by siRNA significant reversed the enhanced glucose uptake, lactate acid production and ECAR by THBS2 overexpression (**Figures 4D, E**, and **S4F**). Moreover, HIF-1α knockdown inhibits the upregulated mRNA expression of *GLUT1, HK2, ALDOA, PKM2*, and *LDHA* by THBS2 overexpression (**Figure 4F**). In addition, HIF-1α knockdown eliminated the promoting cell viability by THBS2 overexpression (**Figure 4G**).

### THBS2 Is a Potential Therapeutic Target for CRC

**Figure 5:**
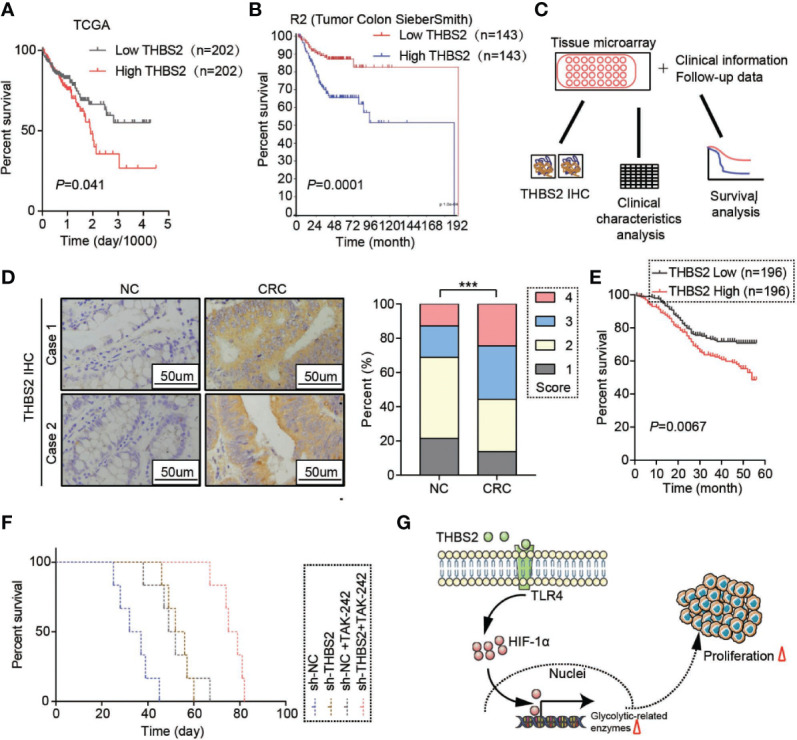
THBS2 is a potential therapeutic target for CRC. **(A)** Overall survival analysis based on the mRNA expression of THBS2 from the TCGA dataset. **(B)** Overall survival analysis based on the mRNA expression of THBS2 from the TCGA dataset. **(C)** Analysis of the protein expression of THBS2 in tissue microarray, linked to clinical information and follow-up data. **(D)** The protein expression of THBS2 in a CRC tissue microarray (392 cases of paired CRC and adjacent non-cancerous tissues) by IHC. **(E)** Overall survival analysis of the protein expression of THBS2, based on the prognostic information of patients with CRC from tissue microarray data. **(F)** Overall survival of the tumor-bearing null mice in the sh-NC, sh-THBS2, sh-NC+TAK-242, and sh-THBS2+TAK-242 group. **(G)** THBS2 interacted with TLR4, which enhanced aerobic glycolysis, and eventually contributed to CRC progress. Measurement data are presented as the mean ± SD. Student’s *t*-test was used for statistical analysis. The Kaplan–Meier method and log-rank test were used for statistical analysis. Spearman’s rank correlation was used to analyze the correlation between THBS2 expression and pathological staging; ***p < 0.001.

Further survival analysis based on TCGA and R2 datasets demonstrated that high mRNA expression of THBS2 predicts a poor prognosis of CRC patients (**Figures 5A, B**). Due to the IHC staining of THBS2 by a tissue microarray containing 392 cases of CRC and paired adjacent colorectal tissues, we found that the protein expression of THBS2 was upregulated in CRC compared with that in the adjacent colorectal tissues (**Figures 5C, D**). The analysis of the clinical characteristics showed that the high protein expression of THBS2 was closely associated with tumor size and its pathological stage (**Table 1**). Combined with follow-up data, CRC patients with high protein expression of THBS2 showed a significantly lower survival rate than those with low protein expression of THBS2 (**Figure 5E**). The *in vivo* experiment indicated that blocking THBS2/TLR4 interaction significantly extends the survival of tumor-bearing mice (**Figure 5F**). All in all, we discovered that THBS2 interacts with TLR4, which enhances aerobic glycolysis, and eventually contributes to CRC progress (**Figure 5G**).

**Table 1:** Correlation between clinical features and THBS expression.

	THBS2		*P value*
	Low (n=196)	High(n=196)	
**Age (year)**	59.51 ± 17.53	60.62 ± 17.29	0.528
**Sex**			
Male	120	112	0.411
Female	76	84	
**Tumor size (cm)**	4.97 ± 2.54	7.09 ± 3.12	**0.0001**
**T stage**			
T1	6	5	0.221
T2	23	13	
T3	50	44	
T4	117	134	
**Lymph node invasion**			
yes	96	87	0.362
no	100	109	
**Distant metastasis**			
yes	40	41	0.901
no	156	155	
**Pathological stage**			
I	22	14	**<0.0001**
Ⅱ	95	58	
Ⅲ	39	83	
Ⅳ	40	41	

Bold value showed statistically significant difference.

## Discussion

In the THBS family, THBS1 has been extensively researched, with diverse functions in tumor progress (16). THBS1 was first reported in breast cancer in which THBS1 promotes the lung metastasis of breast cancer by facilitating cell adhesion to vessel walls (17). However, Isenberg et al. (16) hold the opposing view that THBS1 is an endogenous angiogenesis inhibitor, which increases the apoptosis of tumor cells and restricts tumor growth by blocking NO-driven angiogenesis. Many related studies have consistently illustrated an anti-angiogenesis effect of THBS1 in human cancer (18, 19), although emerging evidence points to a huge transverse. THBS1 aberrant expression *via* TGF-β-smad3 contributes to invasive behavior during glioblastoma (GBM) expansion (20). Therefore, an integral and comprehensive study is needed to probe these controversies to determine the real role of THBS1 in human cancer. Compared with THBS1, researches on THBS2 are limited in human cancer and especially rare in CRC. In early pancreatic ductal adenocarcinoma (PDAC) and in cooperation with CA19-9, THBS2 can be a blood marker for the detection of patients at high risk for PDAC (21). Moreover, THBS2 promotes tumor progress, including cell proliferation, migration, and invasion (22–24). However, it is still unclear whether THBS2 plays a role in regulating CRC development. The analysis of the related data from the TCGA and GEO datasets in this study shows that THBS2 is upregulated in CRC tissues. THBS2 knockdown and overexpression inhibit and promote CRC progress *in vivo* and *in vitro*.

Notably, THBS regulates the tumor cell function *via* interaction with receptors or molecules in the cell surface, including CD36 and CD47 (25, 26). To find the potential receptors or molecules interacting with THSB2 in CRC, we carried out a GSEA analysis and discovered that the TLR pathway is dominantly enriched in the THBS2-high group. TLRs are vital cell surface receptors that play a key role in innate immunity and have crosstalk with adaptive immunity (27, 28). As pattern recognition receptors, TLRs recognize both conserved molecular patterns in microbes (pathogen-associated molecular patterns: PAMPs) and endogenous ligands (danger-associated molecular patterns: DAMPs). In CRC, the role of TLRs is particularly important due to a close association between CRC and intestinal microorganism, including bacteria, fungi, and viruses (29–33). In addition to PAMPs, TLRs also recognize endogenous ligands, such as endogenous RNA or DNA and heat shock proteins (HSPs) (34, 35). TLRs also interact with the other molecules in the cell membrane, including CD36 and EGFR (36–38). The subsequent experiments in our study showed that THBS2 interacts with TLR4 but not with other TLRs and that THBS2/TLR4 promotes CRC cell proliferation. Silencing TLR4 reverses the proliferation-promoted role by THBS2 overexpression.

Extreme energy demand and essential substances synthesis are characteristic alterations in tumor cells, allowing them to maintain continued and rapid proliferation (39, 40). Aerobic glycolysis is a typical change in glucose metabolism in tumor cells. To the best of our knowledge, under normal physiological conditions, human cells underlie oxidative phosphorylation of glucose to produce ATP. Once meeting hypoxia, oxidative phosphorylation is transformed into anaerobic glycolysis to maintain the ATP production. However, this commonsense is no longer correct in tumor cells. Tumor cells tend to exhibit a significantly glycolytic trend, even in normoxia, which was first reported by Warberg (41). During the past decades, numerous studies have focused on this phenomenon and tried to determine its underlying mechanisms. From the energy perspective, related researches confirm that unit glucose provides less ATP through glycolysis and that glycolysis is a quicker way to produce ATP (42). In addition, through the pentose phosphate pathway (PPP)—a bypass of glycolysis—glucose can be transformed into nucleotide, which is essential for tumor cell proliferation (43). Excess lactic acid production, a glycolysis product, plays a pro-inflammatory and anti-immune role in human cancer (44, 45). TLRs have been reported to control glycolysis in immune system cells (46, 47). In this study, we found that THBS2/TLR4 interaction regulates glycolysis, glucose consumption, and lactate production. Hence, THBS2/TLR4 interaction upregulates glycolysis-related genes (*GLUT1, HK2, ALDOA, PKM2*, and *LDHA*) in CRC cells.

Metabolic switch to glycolysis in tumor cells is both controlled by glycolysis-related enzymes and some vital signaling pathways. In this article, we found that the THBS2/TLR4 axis upregulates the protein expression of HIF-1α. HIF-1α inhibition reversed the upregulation of *GLUT1, HK2, ALDOA, PKM2*, and *LDHA* by the THBS2/TLR4 axis. Recent studies have documented that BGN interacting with TLR2/TLR4 promotes the mRNA expression of HIF-1α (48). However, in this study, we found THBS2/TLR4 interaction did not affect mRNA expression of HIF-1α, which indicated THBS2/TLR4 interaction did not affect transcription of HIF-1α mRNA. HIF-1α degradation is regulated by PHDs and we found THBS2/TLR4 interaction also had no effect on PHD1 and PHD2 expression, which indicated THBS2/TLR4 interaction did not involve in HIF-1α degradation. Akt/mTOR/p70S6K/4E-BP1 phosphorylation plays a vital role in regulating HIF-1a expression at the translational level (49). Emerging evidence shows PI3K-Akt signaling pathway is activated rapidly in response to TLRs activation (50). Therefore, THBS2/TLR4 axis might regulate HIF-1a expression at the translational level.

As we known, HIF-1α was a vital transcription factor, which contributed to tumor growth in many solid tumors, including CRC. HIF-1α accumulation contributes to glycolysis by enhancing transcription of glycolysis-related genes, including *GLUT1, HK2, ALDOA, PKM2*, and *LDHA*, which was the most important method for tumor cells to get energy and growth (51, 52). However, several reports that indicate that HIF-1α inhibits cell proliferation (53). These controversial results may be due to different tumor types and models.

In conclusion, we found that THBS2 interacted with TLR4, which enhanced aerobic glycolysis and eventually contributes to CRC progress. This study provides new therapeutic insights for patients with CRC.

## Data Availability Statement

The raw data supporting the conclusions of this article will be made available by the authors, without undue reservation.

## Ethics Statement

The studies involving human participants were reviewed and approved by Research Ethics Committee of Renji Hospital. The patients/participants provided their written informed consent to participate in this study. The animal study was reviewed and approved by Research Ethics Committee of Renji Hospital

## Author Contributions

QX and HX designed the experiment. CX, LG, MK, and SW performed the experiment. All authors contributed to the article and approved the submitted version.

## Funding

Our article was supported by National Natural Science Foundation of China (82072671).

## Conflict of Interest

The authors declare that the research was conducted in the absence of any commercial or financial relationships that could be construed as a potential conflict of interest.

## References

[1] Fillon M Study aims to improve colorectal cancer screening rates. CA: Cancer J Clin (2019) 69(3):161–3. 10.3322/caac.21472 30861095

[2] Wang J Li Y CD36 tango in cancer: signaling pathways and functions. Theranostics (2019) 9(17):4893–908. 10.7150/thno.36037 PMC6691380 31410189

[3] Lee YJ Koch M Karl D Torres-Collado AX Fernando NT Rothrock C Variable inhibition of thrombospondin 1 against liver and lung metastases through differential activation of metalloproteinase ADAMTS1. Cancer Res (2010) 70(3):948–56. 10.1158/0008-5472.CAN-09-3094 PMC2934910 20103648

[4] Huang WT Chong IW Chen HL Li CY Hsieh CC Kuo HF Pigment epithelium-derived factor inhibits lung cancer migration and invasion by upregulating exosomal thrombospondin 1. Cancer Lett (2019) 442:287–98. 10.1016/j.canlet.2018.10.031 30439539

[5] Lee KW Lee SS Hwang JE Jang HJ Lee HS Oh SC Development and Validation of a Six-Gene Recurrence Risk Score Assay for Gastric Cancer. Clin Cancer Res Off J Am Assoc Cancer Res (2016) 22(24):6228–35. 10.1158/1078-0432.CCR-15-2468 PMC5458637 27654712

[6] Denefle T Boullet H Herbi L Newton C Martinez-Torres AC Guez A Thrombospondin-1 Mimetic Agonist Peptides Induce Selective Death in Tumor Cells: Design, Synthesis, and Structure-Activity Relationship Studies. J Med Chem (2016) 59(18):8412–21. 10.1021/acs.jmedchem.6b00781 27526615

[7] Boguslawska J Rodzik K Poplawski P Kedzierska H Rybicka B Sokol E TGF-beta1 targets a microRNA network that regulates cellular adhesion and migration in renal cancer. Cancer Lett (2018) 412:155–69. 10.1016/j.canlet.2017.10.019 29079415

[8] Wei WF Zhou CF Wu XG He LN Wu LF Chen XJ MicroRNA-221-3p, a TWIST2 target, promotes cervical cancer metastasis by directly targeting THBS2. Cell Death Dis (2017) 8(12):3220. 10.1038/s41419-017-0077-5 29242498 PMC5870596

[9] Wu XG Zhou CF Zhang YM Yan RM Wei WF Chen XJ Cancer-derived exosomal miR-221-3p promotes angiogenesis by targeting THBS2 in cervical squamous cell carcinoma. Angiogenesis (2019) 22(3):397–410. 10.1007/s10456-019-09665-1 30993566

[10] Cao M Chen F Xie N Cao MY Chen P Lou Q c-Jun N-terminal kinases differentially regulate TNF- and TLRs-mediated necroptosis through their kinase-dependent and -independent activities. Cell Death Dis (2018) 9(12):1140. 10.1038/s41419-018-1189-2 30442927 PMC6238001

[11] Nunes KP de Oliveira AA Mowry FE Biancardi VC Targeting toll-like receptor 4 signalling pathways: can therapeutics pay the toll for hypertension? Br J Pharmacol (2019) 176(12):1864–79. 10.1111/bph.14438 PMC6534778 29981161

[12] Meng F Wu L Dong L Mitchell AV James Block C Liu J EGFL9 promotes breast cancer metastasis by inducing cMET activation and metabolic reprogramming. Nat Commun (2019) 10(1):5033. 10.1038/s41467-019-13034-3 31695034 PMC6834558

[13] Hoxhaj G Manning BD The PI3K-AKT network at the interface of oncogenic signalling and cancer metabolism. Nat Rev Cancer (2019) 20(2):74–88. 10.1038/s41568-019-0216-7 31686003 PMC7314312

[14] Abbaszadeh Z Cesmeli S Biray Avci C Crucial players in glycolysis: Cancer progress. Gene (2020) 726:144158. 10.1016/j.gene.2019.144158 31629815

[15] Xu C Tian G Jiang C Xue H Kuerbanjiang M Sun L NPTX2 promotes colorectal cancer growth and liver metastasis by the activation of the canonical Wnt/beta-catenin pathway via FZD6. Cell Death Dis (2019) 10(3):217. 10.1038/s41419-019-1467-7 30833544 PMC6399240

[16] Isenberg JS Martin-Manso G Maxhimer JB Roberts DD Regulation of nitric oxide signalling by thrombospondin 1: implications for anti-angiogenic therapies. Nat Rev Cancer (2009) 9(3):182–94. 10.1038/nrc2561 PMC2796182 19194382

[17] Kazerounian S Yee KO Lawler J Thrombospondins in cancer. Cell Mol Life Sci CMLS (2008) 65(5):700–12. 10.1007/s00018-007-7486-z PMC2752021 18193162

[18] Kodama J Hashimoto I Seki N Hongo A Yoshinouchi M Okuda H Thrombospondin-1 and -2 messenger RNA expression in invasive cervical cancer: correlation with angiogenesis and prognosis. Clin Cancer Res Off J Am Assoc Cancer Res (2001) 7(9):2826–31. 11555600

[19] Guerrero D Guarch R Ojer A Casas JM Ropero S Mancha A Hypermethylation of the thrombospondin-1 gene is associated with poor prognosis in penile squamous cell carcinoma. BJU Int (2008) 102(6):747–55. 10.1111/j.1464-410X.2008.07603.x 18336597

[20] Daubon T Leon C Clarke K Andrique L Salabert L Darbo E Deciphering the complex role of thrombospondin-1 in glioblastoma development. Nat Commun (2019) 10(1):1146. 10.1038/s41467-019-08480-y 30850588 PMC6408502

[21] Kim J Bamlet WR Oberg AL Chaffee KG Donahue G Cao XJ Detection of early pancreatic ductal adenocarcinoma with thrombospondin-2 and CA19-9 blood markers. Sci Trans Med (2017) 9(398):eaah5583. 10.1126/scitranslmed.aah5583 PMC5727893 28701476

[22] Tian Q Liu Y Zhang Y Song Z Yang J Zhang J THBS2 is a biomarker for AJCC stages and a strong prognostic indicator in colorectal cancer. J BUON Off J Balkan Union Oncol (2018) 23(5):1331–6. 30570855

[23] Wang X Zhang L Li H Sun W Zhang H Lai M THBS2 is a Potential Prognostic Biomarker in Colorectal Cancer. Sci Rep (2016) 6:33366. 10.1038/srep33366 27632935 PMC5025892

[24] Fei W Chen L Chen J Shi Q Zhang L Liu S RBP4 and THBS2 are serum biomarkers for diagnosis of colorectal cancer. Oncotarget (2017) 8(54):92254–64. 10.18632/oncotarget.21173 PMC5696178 29190912

[25] Lawler PR Lawler J Molecular basis for the regulation of angiogenesis by thrombospondin-1 and -2. Cold Spring Harbor Perspect Med (2012) 2(5):a006627. 10.1101/cshperspect.a006627 PMC3331684 22553494

[26] Chen PC Tang CH Lin LW Tsai CH Chu CY Lin TH Thrombospondin-2 promotes prostate cancer bone metastasis by the up-regulation of matrix metalloproteinase-2 through down-regulating miR-376c expression. J Hematol Oncol (2017) 10(1):33. 10.1186/s13045-017-0390-6 28122633 PMC5264454

[27] Song F Yi Y Li C Hu Y Wang J Smith DE Regulation and biological role of the peptide/histidine transporter SLC15A3 in Toll-like receptor-mediated inflammatory responses in macrophage. Cell Death Dis (2018) 9(7):770. 10.1038/s41419-018-0809-1 29991810 PMC6039463

[28] Xing Y Cao R Hu HM TLR and NLRP3 inflammasome-dependent innate immune responses to tumor-derived autophagosomes (DRibbles). Cell Death Dis (2016) 7(8):e2322. 10.1038/cddis.2016.206 27490927 PMC5108312

[29] Isaza-Correa JM Liang Z van den Berg A Diepstra A Visser L Toll-like receptors in the pathogenesis of human B cell malignancies. J Hematol Oncol (2014) 7:57. 10.1186/s13045-014-0057-5 25112836 PMC4237867

[30] Abreu MT Toll-like receptor signalling in the intestinal epithelium: how bacterial recognition shapes intestinal function. Nat Rev Immunol (2010) 10(2):131–44. 10.1038/nri2707 20098461

[31] Xie L Jiang FC Zhang LM He WT Liu JH Li MQ Targeting of MyD88 Homodimerization by Novel Synthetic Inhibitor TJ-M2010-5 in Preventing Colitis-Associated Colorectal Cancer. J Natl Cancer Institute (2016) 108(4):djv364. 10.1093/jnci/djv364 26712311

[32] Tsoi H Chu ESH Zhang X Sheng J Nakatsu G Ng SC Peptostreptococcus anaerobius Induces Intracellular Cholesterol Biosynthesis in Colon Cells to Induce Proliferation and Causes Dysplasia in Mice. Gastroenterology (2017) 152(6):1419–33 e5. 10.1053/j.gastro.2017.01.009 28126350

[33] Kesselring R Glaesner J Hiergeist A Naschberger E Neumann H Brunner SM IRAK-M Expression in Tumor Cells Supports Colorectal Cancer Progression through Reduction of Antimicrobial Defense and Stabilization of STAT3. Cancer Cell (2016) 29(5):684–96. 10.1016/j.ccell.2016.03.014 27150039

[34] Krieger J Riedl P Stifter K Roman-Sosa G Seufferlein T Wagner M Endogenously Expressed Antigens Bind Mammalian RNA via Cationic Domains that Enhance Priming of Effector CD8 T Cells by DNA Vaccination. Mol Ther J Am Soc Gene Ther (2019) 27(3):661–72. 10.1016/j.ymthe.2019.01.011 PMC6403493 30713086

[35] Muralidharan S Lim A Catalano D Mandrekar P Human Binge Alcohol Intake Inhibits TLR4-MyD88 and TLR4-TRIF Responses but Not the TLR3-TRIF Pathway: HspA1A and PP1 Play Selective Regulatory Roles. J Immunol (2018) 200(7):2291–303. 10.4049/jimmunol.1600924 PMC5860983 29445009

[36] Abe T Shimamura M Jackman K Kurinami H Anrather J Zhou P Key role of CD36 in Toll-like receptor 2 signaling in cerebral ischemia. Stroke (2010) 41(5):898–904. 10.1161/STROKEAHA.109.572552 20360550 PMC2950279

[37] Seimon TA Nadolski MJ Liao X Magallon J Nguyen M Feric NT Atherogenic lipids and lipoproteins trigger CD36-TLR2-dependent apoptosis in macrophages undergoing endoplasmic reticulum stress. Cell Metab (2010) 12(5):467–82. 10.1016/j.cmet.2010.09.010 PMC2991104 21035758

[38] Chattopadhyay S Veleeparambil M Poddar D Abdulkhalek S Bandyopadhyay SK Fensterl V EGFR kinase activity is required for TLR4 signaling and the septic shock response. EMBO Rep (2015) 16(11):1535–47. 10.15252/embr.201540337 PMC4641505 26341626

[39] Cancer M Drews LF Bengtsson J Bolin S Rosen G Westermark B BET and Aurora Kinase A inhibitors synergize against MYCN-positive human glioblastoma cells. Cell Death Dis (2019) 10(12):881. 10.1038/s41419-019-2120-1 31754113 PMC6872649

[40] Zhao SJ Shen YF Li Q He YJ Zhang YK Hu LP SLIT2/ROBO1 axis contributes to the Warburg effect in osteosarcoma through activation of SRC/ERK/c-MYC/PFKFB2 pathway. Cell Death Dis (2018) 9(3):390. 10.1038/s41419-018-0419-y 29523788 PMC5844886

[41] Warburg O On the origin of cancer cells. Science (1956) 123(3191):309–14. 10.1126/science.123.3191.309 13298683

[42] Icard P Shulman S Farhat D Steyaert JM Alifano M Lincet H How the Warburg effect supports aggressiveness and drug resistance of cancer cells? Drug Resist Updates Rev Commentaries Antimicrobial Anticancer Chemother (2018) 38:1–11. 10.1016/j.drup.2018.03.001 29857814

[43] Zhang Q Qin Y Zhao J Tang Y Hu X Zhong W Thymidine phosphorylase promotes malignant progression in hepatocellular carcinoma through pentose Warburg effect. Cell Death Dis (2019) 10(2):43. 10.1038/s41419-018-1282-6 30674871 PMC6426839

[44] Locatelli SL Careddu G Serio S Consonni FM Maeda A Viswanadha S Targeting Cancer Cells and Tumor Microenvironment in Preclinical and Clinical Models of Hodgkin Lymphoma Using the Dual PI3Kdelta/gamma Inhibitor RP6530. Clin Cancer Res Off J Am Assoc Cancer Res (2019) 25(3):1098–112. 10.1158/1078-0432.CCR-18-1133 30352904

[45] Gao F Tang Y Liu WL Zou MZ Huang C Liu CJ Intra/Extracellular Lactic Acid Exhaustion for Synergistic Metabolic Therapy and Immunotherapy of Tumors. Advanced Mater (2019) 2019:e1904639. 10.1002/adma.201904639 31692128

[46] Everts B Amiel E Huang SC Smith AM Chang CH Lam WY TLR-driven early glycolytic reprogramming via the kinases TBK1-IKKvarepsilon supports the anabolic demands of dendritic cell activation. Nat Immunol (2014) 15(4):323–32. 10.1038/ni.2833 PMC4358322 24562310

[47] Mogilenko DA Haas JT L’Homme L Fleury S Quemener S Levavasseur M Metabolic and Innate Immune Cues Merge into a Specific Inflammatory Response via the UPR. Cell (2019) 177(5):1201–16 e19. 10.1016/j.cell.2019.03.018 31031005

[48] Hu L Zang MD Wang HX Li JF Su LP Yan M Biglycan stimulates VEGF expression in endothelial cells by activating the TLR signaling pathway. Mol Oncol (2016) 10(9):1473–84. 10.1016/j.molonc.2016.08.002 PMC5423211 27590684

[49] Mi C Ma J Shi H Li J Wang F Lee JJ 4’,6-dihydroxy-4-methoxyisoaurone inhibits the HIF-1alpha pathway through inhibition of Akt/mTOR/p70S6K/4E-BP1 phosphorylation. J Pharmacol Sci (2014) 125(2):193–201. 10.1254/jphs.13273FP 25075425

[50] McGuire VA Gray A Monk CE Santos SG Lee K Aubareda A Cross talk between the Akt and p38alpha pathways in macrophages downstream of Toll-like receptor signaling. Mol Cell Biol (2013) 33(21):4152–65. 10.1128/MCB.01691-12 PMC3811899 23979601

[51] Briggs KJ Koivunen P Cao S Backus KM Olenchock BA Patel H Paracrine Induction of HIF by Glutamate in Breast Cancer: EglN1 Senses Cysteine. Cell (2016) 166(1):126–39. 10.1016/j.cell.2016.05.042 PMC4930557 27368101

[52] Shukla SK Purohit V Mehla K Gunda V Chaika NV Vernucci E MUC1 and HIF-1alpha Signaling Crosstalk Induces Anabolic Glucose Metabolism to Impart Gemcitabine Resistance to Pancreatic Cancer. Cancer Cell (2017) 32(1):71–87 e7. 10.1016/j.ccell.2017.06.004 28697344 PMC5533091

[53] Melendez-Rodriguez F Urrutia AA Lorendeau D Rinaldi G Roche O Bogurcu-Seidel N HIF1alpha Suppresses Tumor Cell Proliferation through Inhibition of Aspartate Biosynthesis. Cell Rep (2019) 26(9):2257–65 e4. 10.1016/j.celrep.2019.01.106 30811976

