# Supplementary material for: Thrombospondin 2/Toll-Like Receptor 4 Axis Contributes to HIF-1α-Derived Glycolysis in Colorectal Cancer
Source: Front Oncol. 2020 Nov 10;10:557730. doi: 10.3389/fonc.2020.557730 (PMC7683806; doi:10.3389/fonc.2020.557730)
Supplement: Supplementary file 1 [file DataSheet_1.docx]

**Supplementary figure legend**

**Figure S1. Knockdown and overexpression of THBS2 promotes colony formation *in vitro*.** (A) THBS2 knockdown in LoVo cells by shRNA. (B) THBS2 knockdown in RKO cells by shRNA. (C) THBS2 overexpression in SW620 cells by lentivirus transfection. (D) Colony formation ability of LoVo cells transfected with sh-THBS2 or sh-control, as analyzed with colony formation assay. (E) Colony formation ability of RKO cells transfected with sh-THBS2 or sh-control, as analyzed with colony formation assay. (F) Colony formation ability of SW620 cells transfected with Vector or THBS2-OV, as analyzed with colony formation assay. All experiments were performed in triplicate. Measurement data are presented as the mean ± SD. Student’s *t*-test was used for statistical analysis. Ns. represents no statistical difference; ****p* < 0.001.

**Figure S2. Other TLRs not involved in CRC proliferation by THBS2.** (A) Effect of THBS2 knockdown on TLRs expression in LoVo cells. (B) Effect of THBS2 knockdown on TLRs expression in RKO cells. (C) Effect of THBS2 overexpression on TLRs expression in SW620 cells by lentivirus transfection. (D) Viability of THBS2-OV SW620 cells transfected with si-control and si-TLR1; (E) Viability of THBS2-OV SW620 cells transfected with si-control and si-TLR2; (F) Viability of THBS2-OV SW620 cells transfected with si-control and si-TLR3; (G) Viability of THBS2-OV SW620 cells transfected with si-control and si-TLR5; (H) Viability of THBS2-OV SW620 cells transfected with si-control and si-TLR6; (I) Viability of THBS2-OV SW620 cells transfected with si-control and si-TLR7; (J) Viability of THBS2-OV SW620 cells transfected with si-control and si-TLR8; (K) Viability of THBS2-OV SW620 cells transfected with si-control and si-TLR9; (L) Viability of THBS2-OV SW620 cells transfected with si-control and si-TLR10. Measurement data are presented as the mean ± SD. Student’s *t*-test was used for statistical analysis.

**Figure S3. THBS2/TLR4 interaction plays a role in glycolysis in CRC cells.** (A) O2 consumption rate (OCR) of LoVo or RKO cells in the sh-NC and sh-THBS2 group was detected via a Seahorse Bioscience XFp analyzer. (B) O2 consumption rate (OCR) of SW620 or SW480 cells in the Vector and THBS2-OV group with the treatment of siTLR4 or TAK-242 was detected via a Seahorse Bioscience XFp analyzer. O: Oligomycin, F: FCCP, A&R: antimycin A/rotenone. (C) The glucose uptake of LoVo or RKO cells in the sh-NC and sh-THBS2 group. (D) Lactic acid formation of LoVo or RKO cells in the sh-NC and sh-THBS2 group. (E) mRNA expression of relative genes in the glycolysis of LoVo cells in the sh-NC and sh-THBS2 group. (F) mRNA expression of relative genes in glycolysis of RKO cells in the sh-NC and sh-THBS2 group. (G) THBS2/TLR4 axis upregulated the protein expression of GLUT1, HK2, ALDOA, PKM2, and LDHA (H) THBS2/TLR4 axis affects *GLUT1, HK2, ALDOA, PKM2,* and *LDHA* expression in CRC cells under hypoxia. Measurement data are presented as the mean ± SD. Student’s *t*-test was used for statistical analysis; **p* < 0.05.

**Figure S4. THBS2/TLR4 contributes to glycolysis by HIF-1α.** (A) Effect of THBS2/TLR4 axis on *GLUT1, HK2, ALDOA, PKM2,* and *LDHA* expression in tumor tissue by LoVo cells. (B) Effect of THBS2/TLR4 axis on *GLUT1, HK2, ALDOA, PKM2,* and *LDHA* expression in tumor tissue by SW620 cells. (C) Effect of THBS2/TLR4 axis on *HIF-1α, PHD1, PHD2* and *PHD3* expression by LoVo cells. (D) Effect of THBS2/TLR4 axis on *HIF-1α, PHD1, PHD2* and *PHD3* expression by SW620 cells. (E) Correlation of HIF-1α and THBS2 in tissue microarray. (F) Effect of HIF-1α on ECAR of SW620 and SW480 cellls, which included Vector, THBS2-OV and THBS2-OV+ siHIF-1α groups. Measurement data are presented as the mean ± SD. Student’s *t*-test was used for statistical analysis; **p* < 0.05. ns. represents no statistical difference.
