# Supplementary figures and images for: Thrombospondin 2/Toll-Like Receptor 4 Axis Contributes to HIF-1α-Derived Glycolysis in Colorectal Cancer
Source: Front Oncol. 2020 Nov 10;10:557730. doi: 10.3389/fonc.2020.557730 (PMC7683806; doi:10.3389/fonc.2020.557730)

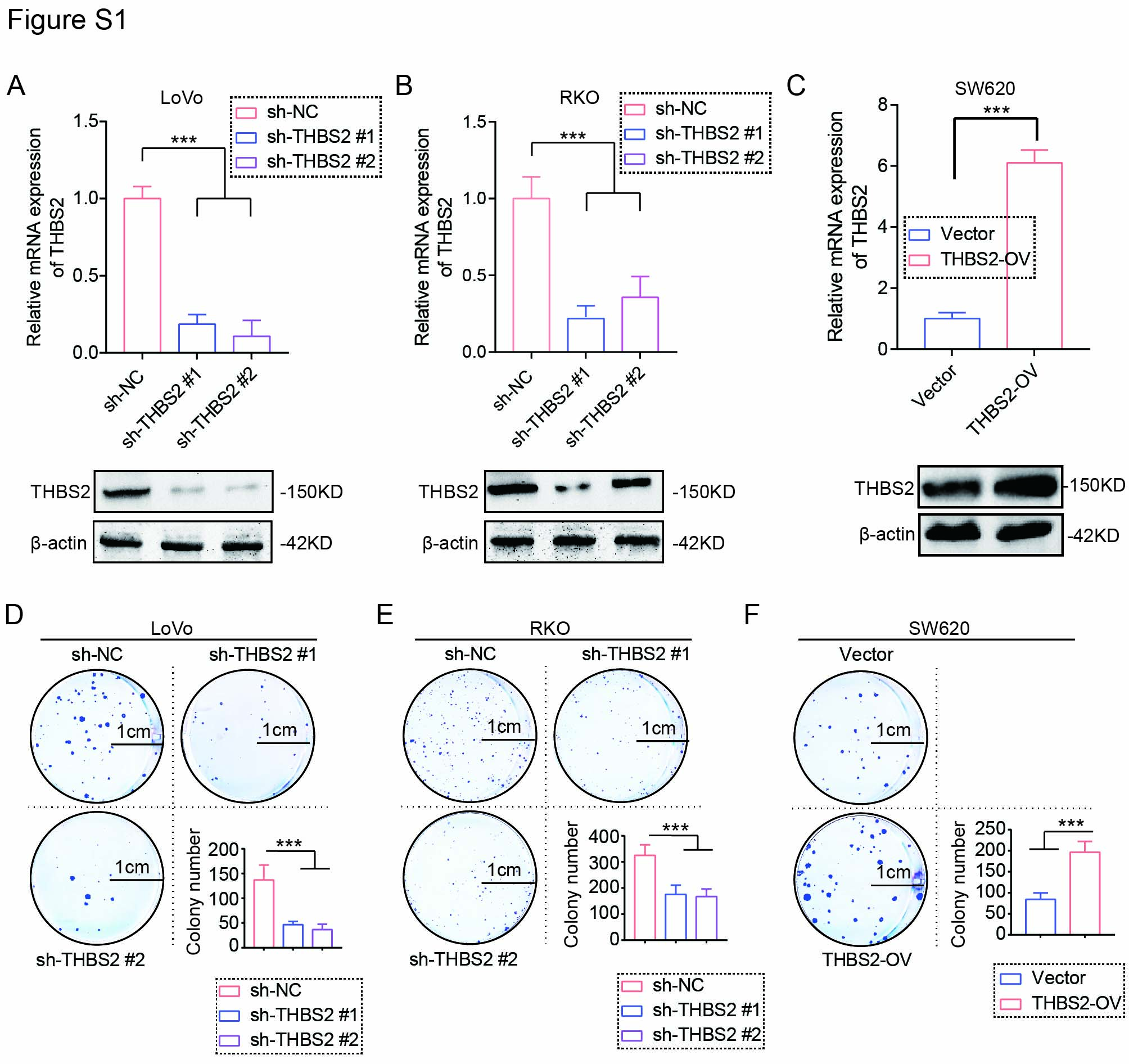

Supplement: Supplementary file 2 [file Image_1.jpeg]

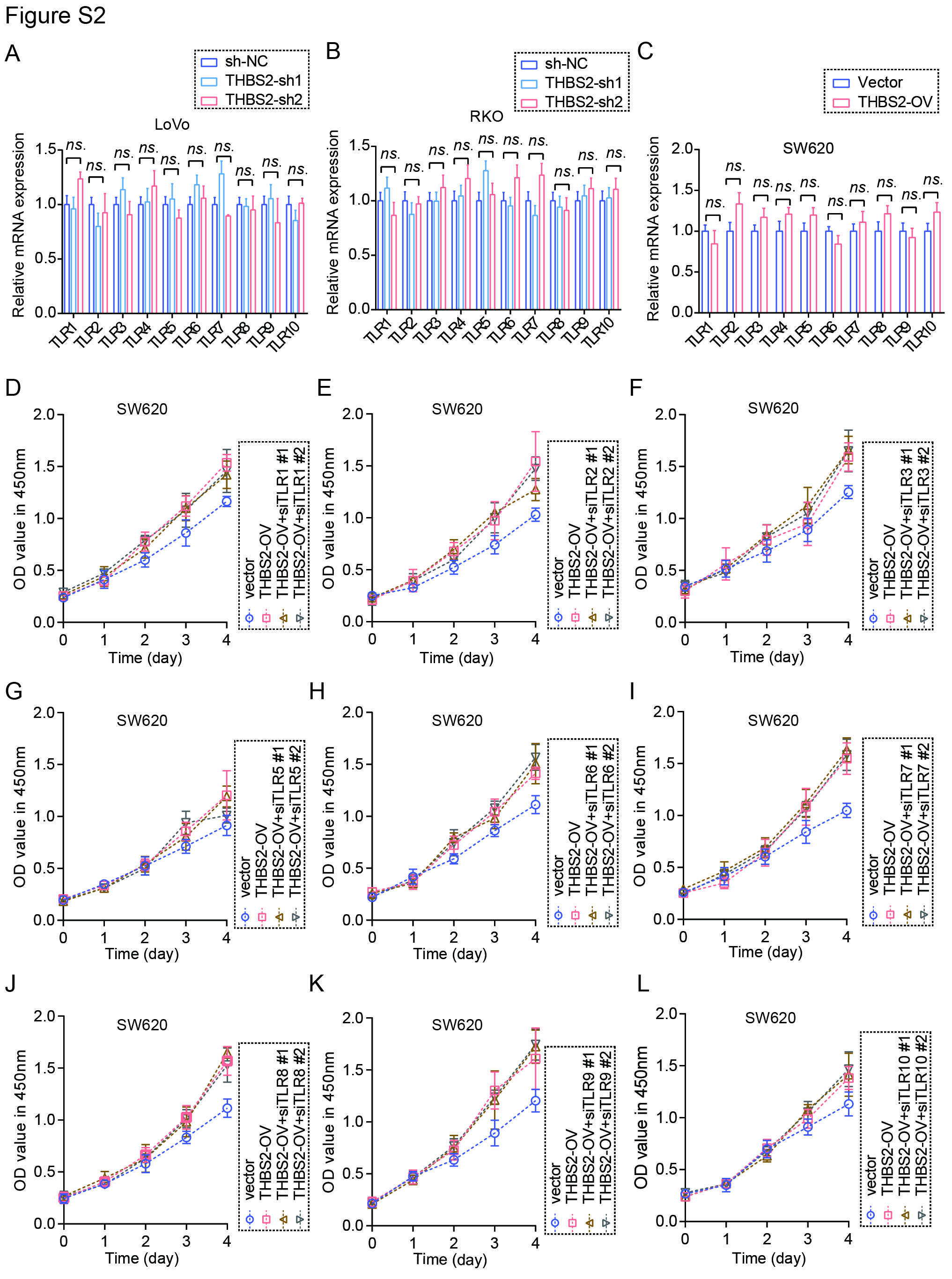

Supplement: Supplementary file 3 [file Image_2.jpeg]

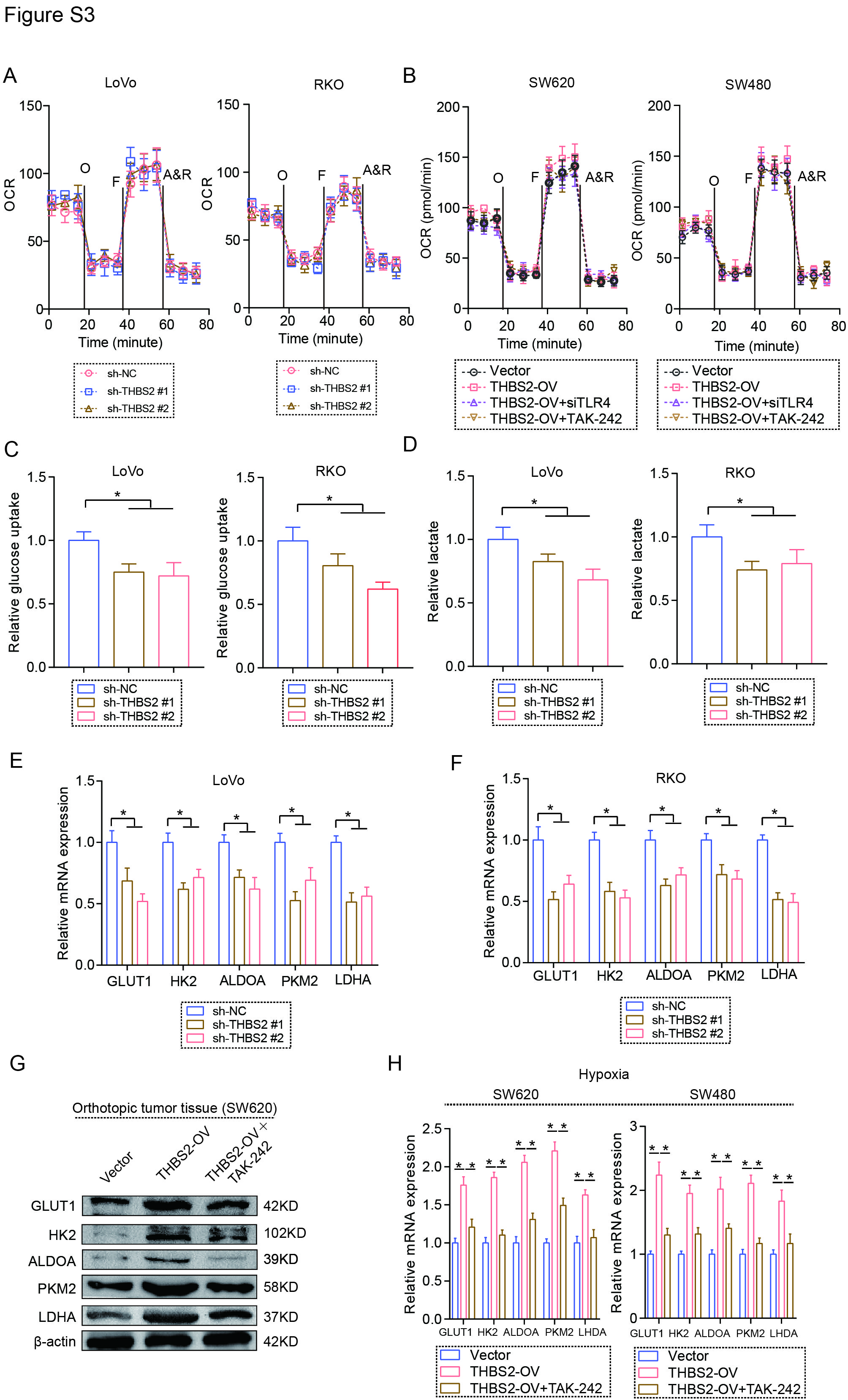

Supplement: Supplementary file 4 [file Image_3.jpeg]

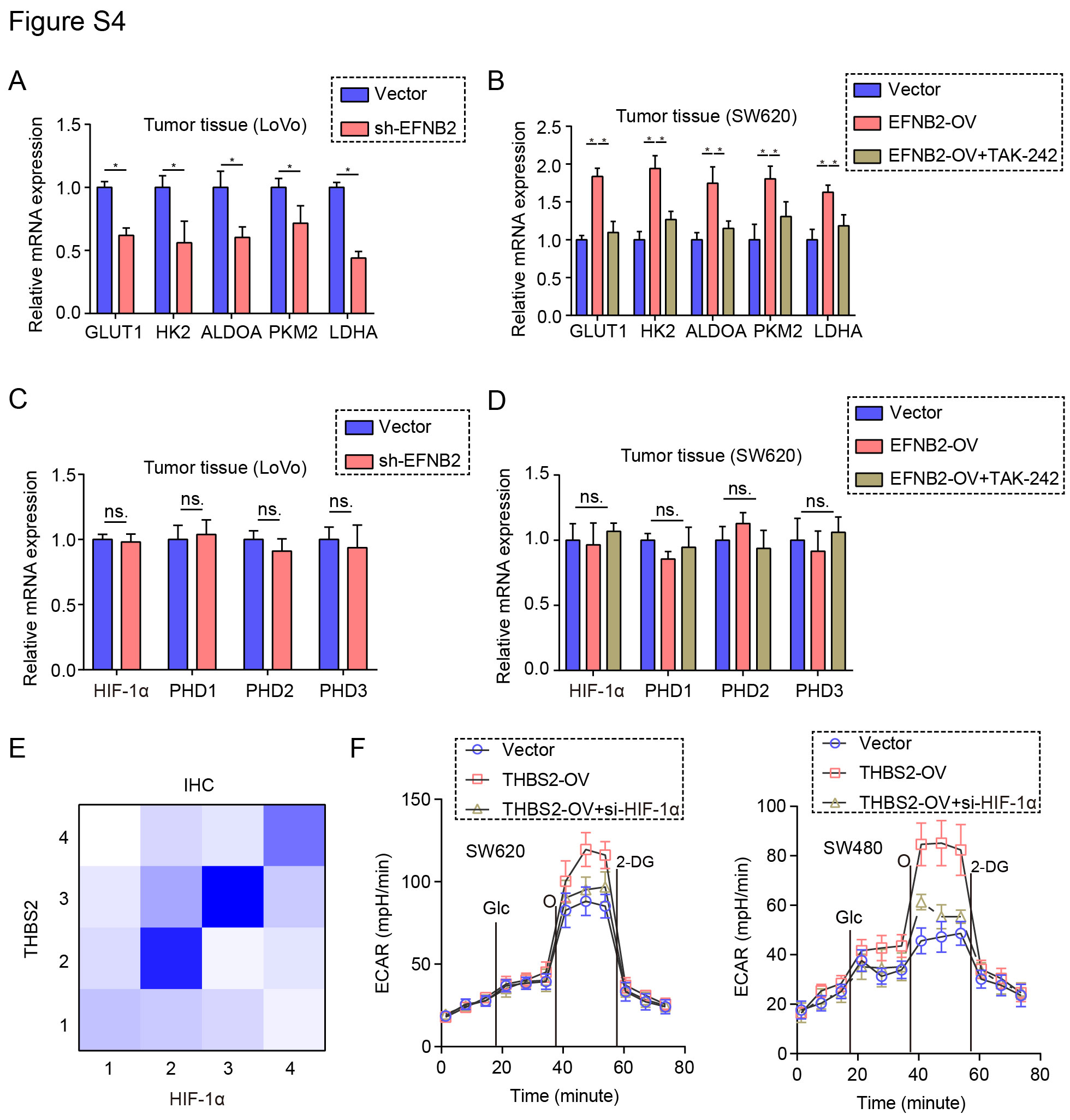

Supplement: Supplementary file 5 [file Image_4.jpeg]
