# Supplementary material for: Thrombospondin 2/Toll-Like Receptor 4 Axis Contributes to HIF-1α-Derived Glycolysis in Colorectal Cancer
Source: Front Oncol. 2020 Nov 10;10:557730. doi: 10.3389/fonc.2020.557730 (PMC7683806; doi:10.3389/fonc.2020.557730)
Supplement: Supplementary file 6 [file Table_1.docx]

**Table S1. primers and sequence used in this study**

|  | | **Sequence** |
| --- | --- | --- |
|  |  |  |
| **TLR1 siNC** | **Sense** | 5'-UUCUUCGAACGUGUCACGUTT-3' |
|  | **Antisense** | 5'-ACGUGACACGUUCGGAGAATT-3' |
| **TLR1 si1** | **Sense** | 5'-GAGCUUUGGACUUCUGACAUCUUAU-3' |
|  | **Antisense** | 5'-AUAAGAUGUCAGAAGUCCAAAGCUC-3' |
| **TLR1 si2** | **Sense** | 5'-CACAACAAGUUGGUGAAGAUUUCUU-3' |
|  | **Antisense** | 5'-AAGAAAUCUUCACCAACUUGUUGUG-3' |
| **TLR2 siNC** | **Sense** | 5'-UUCUCCGAACGUGUCACGUTT-3' |
|  | **Antisense** | 5'-ACGUGACACGUUCGGAGAATT-3' |
| **TLR2 si1** | **Sense** | 5'-CCAGUGUUUGGUGUUGCAAGCAGGA-3' |
|  | **Antisense** | 5'-UCCUGCUUGCAACACCAAACACUGG-3' |
| **TLR2 si2** | **Sense** | 5'-CAGGAGCUCUUAGUGACCAAGUGAA-3' |
|  | **Antisense** | 5'-UUCACUUGGUCACUAAGAGCUCCUG-3' |
| **TLR3 siNC** | **Sense** | 5'-UUCUCCGAACGUGUCACGUTT-3' |
|  | **Antisense** | 5'-ACGUGACACGUUCGGAGAATT-3' |
| **TLR3 si1** | **Sense** | 5'-GAUGUAGGAUUUAACACCAUCUCAA-3' |
|  | **Antisense** | 5'-UUGAGAUGGUGUUAAAUCCUACAUC-3' |
| **TLR3 si2** | **Sense** | 5'-CGAAUUUGACUGAACUCCAUCUCAU-3' |
|  | **Antisense** | 5'-AUGAGAUGGAGUUCAGUCAAAUUCG-3' |
| **TLR4 siNC** | **Sense** | 5'-UUCUCCGAACGUGUCACGUTT-3' |
|  | **Antisense** | 5'-ACGUGACACGUUCGGAGAATT-3' |
| **TLR4 si1** | **Sense** | 5'-AGGCUGACAUUGGCCUCCUUCCUAU-3' |
|  | **Antisense** | 5'-AUAGGAAGGAGGCCAAUGUCAGCCU-3' |
| **TLR4 si2** | **Sense** | 5'-CCACACCCAAUUGCUUCAUGCUUAA-3' |
|  | **Antisense** | 5'-UUAAGCAUGAAGCAAUUGGGUGUGG-3' |
| **TLR5 siNC** | **Sense** | 5'-UUCUCCGAACGUGUCACGUTT-3' |
|  | **Antisense** | 5'-ACGUGACACGUUCGGAGAATT-3' |
| **TLR5 si1** | **Sense** | 5'-AGGCUCCUGCUGAGCUUCAACUAUA-3' |
|  | **Antisense** | 5'-UAUAGUUGAAGCUCAGCAGGAGCCU-3' |
| **TLR5si2** | **Sense** | 5'-CAUCCUUCAUUUGGGAAGUUGAAUU-3' |
|  | **Antisense** | 5'-AAUUCAACUUCCCAAAUGAAGGAUG-3' |
| **TLR6 siNC** | **Sense** | 5'-UUCUCCGAACGUGUCACGUTT-3' |
|  | **Antisense** | 5'-ACGUGACACGUUCGGAGAATT-3' |
| **TLR6 si1** | **Sense** | 5'-CCAGUCUCUUCUGGUUUAAUCAUUA-3' |
|  | **Antisense** | 5'-UAAUGAUUAAACCAGAAGAGACUGG-3' |
| **TLR6 si2** | **Sense** | 5'-UCGGCCAAAUUUAUUUGCAGCUGGA-3' |
|  | **Antisense** | 5'-UCCAGCUGCAAAUAAAUUUGGCCGA-3' |
| **TLR7 siNC** | **Sense** | 5'-UUCUCCGAACGUGUCACGUTT-3' |
|  | **Antisense** | 5'-ACGUGACACGUUCGGAGAATT-3' |
| **TLR7 si1** | **Sense** | 5'-GGACACUGAAGAGACAAAUUCUUAU-3' |
|  | **Antisense** | 5'-AUAAGAAUUUGUCUCUUCAGUGUCC-3' |
| **TLR7 si2** | **Sense** | 5'-CCAGAAGCUUUAGUGGACUCACUUA-3' |
|  | **Antisense** | 5'-UAAGUGAGUCCACUAAAGCUUCUGG-3' |
| **TLR8 siNC** | **Sense** | 5'-UUCUCCGAACGUGUCACGUTT-3' |
|  | **Antisense** | 5'-ACGUGACACGUUCGGAGAATT-3' |
| **TLR8 si1** | **Sense** | 5'-CAGAGUGCAGCAAUCGUCGACUACA-3' |
|  | **Antisense** | 5'-UGUAGUCGACGAUUGCUGCACUCUG-3' |
| **TLR8 si2** | **Sense** | 5'-CAGGAAGAUUAAUGCUGCCUGGUUU-3' |
|  | **Antisense** | 5'-AAACCAGGCAGCAUUAAUCUUCCUG-3' |
| **TLR9 siNC** | **Sense** | 5'-UUCUCCGAACGUGUCACGUTT-3' |
|  | **Antisense** | 5'-ACGUGACACGUUCGGAGAATT-3' |
| **TLR9 si1** | **Sense** | 5'-CCCGCUACUGGUGCUAUCCAGAAUU-3' |
|  | **Antisense** | 5'-AAUUCUGGAUAGCACCAGUAGCGGG-3' |
| **TLR9 si2** | **Sense** | 5'-GGUGAAGGAGCUGUCUGCCAUUUGA-3' |
|  | **Antisense** | 5'-UCAAAUGGCAGACAGCUCCUUCACC-3' |
| **TLR10 siNC** | **Sense** | 5'-UUCUCCGAACGUGUCACGUTT-3' |
|  | **Antisense** | 5'-ACGUGACACGUUCGGAGAATT-3' |
| **TLR10 si1** | **Sense** | 5'-GGUUGGAUUACAGGGAGCAUUUGAU-3' |
|  | **Antisense** | 5'-AUCAAAUGCUCCCUGUAAUCCAACC-3' |
| **TLR10 si2** | **Sense** | 5'-CAGGGAGCAUUUGAUUUCUAUGUUG-3' |
|  | **Antisense** | 5'-CAACAUAGAAAUCAAAUGCUCCCUG-3' |
| **THBS2 shNC** | **Sense** | 5'-TTCTCCGAACGTGTCACGT-3' |
|  |  |  |
| **THBS2 sh1** | **Sense** | 5'- GCTTCGTGCGCTTTGACTACA-3' |
|  |  |  |
| **THBS2 sh2** | **Sense** | 5'- GCAAGATCACCAAGATCATGC-3' |
|  |  |  |
| **THBS2** | **Forward** | 5’- GACACGCTGGATCTCACCTAC-3’ |
|  | **Reverse** | 5’-GAAGCTGTCTATGAGGTCGCA-3’ |
| **18S** | **Forward** | 5’-TGCGAGTACTCAACACCAACA-3’ |
|  | **Reverse** | 5’-GCATATCTTCGGCCCACA-3’ |
| **TLR1** | **Forward** | 5’-CCACGTTCCTAAAGACCTATCCC-3’ |
|  | **Reverse** | 5’-CCAAGTGCTTGAGGTTCACAG-3’ |
| **TLR2** | **Forward** | 5’- TTATCCAGCACACGAATACACAG -3’ |
|  | **Reverse** | 5’-AGGCATCTGGTAGAGTCATCAA-3’ |
| **TLR3** | **Forward** | 5’- TGTCTGGAAGAAAGGGACTTTG-3’ |
|  | **Reverse** | 5’-ACTGCATGATGTACCTTGAATCT-3’ |
| **TLR4** | **Forward** | 5’-AGACCTGTCCCTGAACCCTAT-3’ |
|  | **Reverse** | 5’-CGATGGACTTCTAAACCAGCCA-3’ |
| **TLR5** | **Forward** | 5’- TCCAGGGTTCAAGCGATTC-3’ |
|  | **Reverse** | 5’- CGTTGTCAGTAGCATCAGGAG-3’ |
| **TLR6** | **Forward** | 5’-TGGACTCATATCAAGATGCTCTG-3’ |
|  | **Reverse** | 5’-GTCGGAGAACTGGATTCTGG-3’ |
| **TLR7** | **Forward** | 5’-TCGTGGACTGCACAGACAAG-3’ |
|  | **Reverse** | 5’-GGTATGTGGTTAATGGTGAGGGT -3’ |
| **TLR8** | **Forward** | 5’-ATGTTCCTTCAGTCGTCAATGC-3’ |
|  | **Reverse** | 5’-TTGCTGCACTCTGCAATAACT-3’ |
| **TLR9** | **Forward** | 5’-CTATAACCGGAACTTCTGCCAG-3’ |
|  | **Reverse** | 5’-CTGCTCTGTGTCAGGTGTG-3’ |
| **TLR10** | **Forward** | 5’-CATATAGAGGCAGAAGGACAGG-3’ |
|  | **Reverse** | 5’-GAGAAGCATAATGGACCTTTGG-3’ |
| **HIF-1α** | **Forward** | 5’-ATCCATGTGACCATGAGGAAATG-3’ |
|  | **Reverse** | 5’-TCGGCTAGTTAGGGTACACTTC-3’ |
| **GLUT1** | **Forward** | 5’-TCATCGTGGCTGAACTCTTC-3’ |
|  | **Reverse** | 5’-GATGAAGACGTAGGGACCAC-3’ |
| **HK2** | **Forward** | 5’-GAGCCACCACTCACCCTACT -3’ |
|  | **Reverse** | 5’-CCAGGCATTCGGCAATGTG-3’ |
| **ALDOA** | **Forward** | 5’-ATGCCCTACCAATATCCAGCA-3’ |
|  | **Reverse** | 5’-GCTCCCAGTGGACTCATCTG-3’ |
| **PKM2** | **Forward** | 5’-ATGTCGAAGCCCCATAGTGAA-3’ |
|  | **Reverse** | 5’-TGGGTGGTGAATCAATGTCCA -3’ |
| **LDHA** | **Forward** | 5’-TTGACCTACGTGGCTTGGAAG-3’ |
|  | **Reverse** | 5’-GGTAACGGAATCGGGCTGAAT-3’ |
|  |  |  |
|  |  |  |
|  |  |  |
|  |  |  |
|  |  |  |
|  |  |  |
|  |  |  |
|  |  |  |
